# Editorial for the Special Issue “Polymeric Hydrogels for Biomedical Application”

**DOI:** 10.3390/gels11040221

**Published:** 2025-03-21

**Authors:** Ion Călina, Muhammad Asim Raza

**Affiliations:** 1 National Institute for Laser, Plasma and Radiation Physics, 409 Atomiștilor, 077125 Măgurele, Romania; 2 School of Chemical Engineering, Yeungnam University, 280 Daehak-ro, Gyeongsan 38541, Republic of Korea

Three-dimensional networks of hydrophilic polymers, known as hydrogels, have shown great promise across various fields due to their ability to absorb and retain large amounts of water. Hydrogels can be composed of natural polymers, synthetic polymers, or hybrid materials. Hydrogel-based biomaterials, valued for their excellent degradability, biocompatibility, tunable mechanical properties, and injectability, have gained increasing attention from researchers and clinicians. Crosslinking plays a crucial role in forming the network structure and enhancing the mechanical properties of hydrogels. These materials can be fabricated using either physical or chemical crosslinking mechanisms, which involve physical interaction or covalent bonds between polymer chains, resulting in a more stable and robust network. In this Special Issue, we aim to publish articles that address current challenges and propose innovative solutions in various biomedical areas, including drug delivery, diagnostics, spinal surgery, and oral absorbents.

This Special Issue comprises four research articles and two review articles that explore various aspects of hydrogels for biomedical applications, covering all the aforementioned topics, as follows:

1. The review “Multifunctional Hydrogel Microneedles (HMNs) in Drug Delivery and Diagnostics” by Omidian et al. [1] examines the key materials, fabrication strategies, biosensing capabilities, and drug delivery mechanisms of microneedles. Hydrogel microneedles represent a significant advancement in biosensing and transdermal drug delivery, offering precision drug release, biocompatibility, and non-invasive biomarker monitoring. However, their clinical translation is hindered by scalability challenges, regulatory barriers, and the need for extensive safety validation in human trials. Overcoming these obstacles will be crucial for advancing hydrogel microneedles into clinical practice, paving the way for next-generation patient-centered diagnostics and therapeutics.
2. The article “Injectable and In Situ Phospholipid-Based Phase Separation Gel for Sustained Delivery of Altrenogest” by Li et al. [2] presents the development of soybean phospholipid/caprylic/capric triglyceride-based phase separation injectable gels incorporating altrenogest to achieve efficient and sustained in vivo and in vitro release. The viscosity of all the prepared gels increases upon contact with physiological saline. The incorporation of altrenogest into the gels promotes rapid gel formation through a sol–gel transition mechanism. In vitro release studies and pharmacokinetic profiles of the gels demonstrate promising sustained release, highlighting their potential in pig farming.
3. The article “Crosslinking by Click Chemistry of Hyaluronan Graft Copolymers Involving Resorcinol-Based Cinnamate Derivatives Leading to Gel-like Materials” by Saletti et al. [3] describes the synthesis of graft copolymers of hyaluronic acid containing resorcinol-based cinnamate through a click chemistry approach. The reaction is conducted under very mild conditions, and the resulting series of crosslinked gel-like materials are thoroughly analyzed in terms of their rheological and structural characteristics to assess their potential as viscosupplements. The resistance to hyaluronidase and the presence of a crosslinked structure ensures a longer residence time. The results indicate that the synthesized materials exhibit the desired shear-thinning behavior, optimal swelling performance, and coefficient of friction values comparable to commercial HA solutions used as viscosupplements.
4. The article “Improvement of Adsorption Capacity by Refined Encapsulating Method of Activated Carbon into the Hollow-Type Spherical Bacterial Cellulose Gels for Oral Absorbent” by Hirai et al. [4] presents an improved encapsulation method for incorporating activated carbon into hollow-type spherical bacterial cellulose gels to enhance adsorption capacity for oral absorbents. This process involves sterilization and dissolution treatment using aqueous sodium hydroxide while evaluating adsorption capacity and pH tolerance. The method effectively eliminates concerns regarding the presence of *K. xylinus* in the bacterial cellulose membrane. The study measures the indole saturation adsorption capacity of the obtained gel, demonstrating that the activated carbon prepared using this new method achieves a higher saturated adsorption amount for indoles compared to previously reported approaches.
5. The article “The Influence of the Structural Architecture on the Swelling Kinetics and the Network Behavior of Sodium-Alginate-Based Hydrogels Cross-Linked with Ionizing Radiation” by Călina et al. [5] explores the fabrication of sodium alginate–co-acrylic acid–poly(ethylene) oxide hydrogels crosslinked via electron beam irradiation. The study investigates the effects of the hydrogel diameter, architecture, and composition on the absorption capacities and swelling kinetics. The results indicate that the crosslinked hydrogels exhibit a high gel fraction, improved physicochemical properties, and maximum swelling rate. Based on these findings, the developed hydrogels show potential applications in water purification, particularly for removing dyes, metals, and pharmaceuticals, as well as in systems for the controlled release of bioactive compounds and water retention.
6. The review “Polymeric Dural Biomaterials in Spinal Surgery: A Review” by Yan et al. [6] discusses polymer-based dural biomaterials with potential application in spinal surgery. This review focuses on the fabrication strategies for natural and synthetic polymeric biomaterials to prevent epidural fibrosis. It explores their physicochemical properties and their ability to mitigate excessive fibroblast proliferation. Natural polymers such as hyaluronic acid, chitosan, and collagen are discussed alongside synthetic polymers like poly(lactic-co-glycolic) acid, polycaprolactone, and polyethylene glycol. The review also addresses the challenges that must be overcome to translate laboratory research into clinical practice, as well as the latest advancements in this field.

Ongoing research on hydrogels for biomedical applications promises groundbreaking advancements in areas such as drug delivery, wound healing, regenerative medicine, and tissue engineering. We express our gratitude to the authors for their valuable contributions to this Special Issue and look forward to the transformative impact of their work.
